# Trial of ultrasound guided carpal tunnel release versus traditional open release (TUTOR)

**DOI:** 10.1097/MD.0000000000030775

**Published:** 2022-10-14

**Authors:** Kyle R. Eberlin, Christopher J. Dy, Mark D. Fischer, James L. Gluck, F. Thomas D. Kaplan, Thomas J. McDonald, Larry E. Miller, Alexander Palmer, Marc E. Walker, James F. Watt

**Affiliations:** a Massachusetts General Hospital, Boston, MA, USA; b Washington University, St. Louis, MO, USA; c Twin Cities Orthopedics, Plymouth, MN, USA; d Kansas Orthopaedic Center, Wichita, KS, USA; e Indiana Hand to Shoulder, Indianapolis, IN, USA; f Sierra Orthopedic Institute, Sonora, CA, USA; g Miller Scientific, Johnson City, TN, USA; h Sano Orthopedics, Lee’s Summit, MO, USA; i University of Mississippi Medical Center, Jackson, MS, USA; j Orthopedic Associates, Fort Walton Beach, FL, USA.

**Keywords:** carpal tunnel release, carpal tunnel syndrome, randomized controlled trial, TUTOR, ultrasound

## Abstract

Carpal tunnel release (CTR) is a surgical treatment option for patients with carpal tunnel syndrome (CTS) symptoms that are unresponsive to conservative treatment. Most patients experience symptomatic relief after CTR regardless of the surgical technique. However, direct comparisons of the safety and effectiveness between CTR surgical techniques are limited. The purpose of this randomized controlled trial is to compare the safety and effectiveness of CTR with ultrasound guidance (CTR-US) versus mini-open CTR (mOCTR) in subjects with symptomatic CTS.

**Design and methods:**

TUTOR (Trial of Ultrasound guided CTR versus Traditional Open Release) is a randomized controlled trial in which 120 subjects at up to 12 sites in the United States will be randomized (2:1) to receive CTR-US or mOCTR. The primary endpoint of the study is the percentage of patients who return to normal daily activities within 3 days of the procedure. Secondary endpoints of the study are median time to return to normal daily activities, percentage of patients who return to work within 3 days of the procedure, median time to return to work, Boston Carpal Tunnel Questionnaire Symptom Severity Scale (BCTQ-SSS) change score at 3 months, BCTQ Functional Status Scale (BCTQ-FSS) change score at 3 months, Numeric Pain Scale change score at 3 months, EuroQoL-5 Dimension 5-Level (EQ-5D-5L) change score at 3 months, and the incidence of device- or procedure-related adverse events at 3 months. Patient follow-up in this trial will continue for 1 year.

**Ethics and dissemination:**

This study was approved by a central institutional review board and ongoing trial oversight will be provided by a data safety monitoring board (DSMB). The authors intend to report the results of this trial at medical conferences and peer-reviewed journals. The outcomes of TUTOR will have important clinical and economic implications for all stakeholders involved in treating patients with CTS.

**Study registration:**

ClinicalTrials.gov (https://clinicaltrials.gov): NCT05405218.

**Level of evidence:**

1

## 1. Introduction

Carpal tunnel syndrome (CTS) is the most common peripheral compression neuropathy, affecting approximately 5% of the population.^[1]^ A multitude of treatments are available to treat CTS including activity modification, bracing/splinting, hand therapy, modalities (e.g., therapeutic lasers or ultrasound, iontophoresis), acupuncture, corticosteroid injections, and carpal tunnel release (CTR) surgery.^[2–7]^ Currently, there is no universally accepted algorithm to guide treatment for patients suffering from CTS. The American Academy of Orthopedic Surgery CTS Clinical Practice Guidelines reported that only 3 treatments are strongly supported in the literature: splinting, corticosteroid injections, and CTR.^[4]^ Although some patients with mild to moderate symptoms are successfully treated with splinting and/or corticosteroid injections, those with progressive, refractory, or severe symptoms often undergo CTR for definitive management.^[2,3,5–9]^

The goal of CTR is to reduce pressure on the median nerve by dividing the transverse carpal ligament (TCL) while avoiding iatrogenic injury to surrounding neurovascular structures. Among approximately 600,000 CTR procedures performed in the United States annually,^[1,10]^ most (70%–80%) use an open technique (OCTR) during which a palmar incision is made to dissect down to the TCL and transect it using a scalpel, scissors, or a similar cutting device.^[11–13]^ The standard OCTR technique requires a relatively large incision of 3 to 5 cm and may be associated with a prolonged recovery period due to palmar pain and the need to protect the wound.^[12,14–17]^

Over time, there has been a trend to use smaller incisions (1–3 cm) to reduce surgical morbidity using mini-OCTR (mOCTR) or endoscopic CTR.^[11,16–18]^ Because long-term outcomes and complication profiles are generally equivalent among these CTR procedures,^[19]^ factors related to patient recovery time such as time to return to normal activities and work absenteeism are important considerations that may assist in shared treatment decision-making between physicians and patients.

In recent years, multiple studies have demonstrated the feasibility of using ultrasound to perform CTR through even smaller incisions while maintaining or improving visualization of the carpal tunnel region, including its at-risk neurovascular structures. During CTR using ultrasound guidance (CTR-US), the carpal tunnel is typically accessed through a single small wrist or palmar incision less than 5 mm length and the TCL is transected using a small knife or similar cutting instrument while the carpal tunnel structures are monitored using ultrasound during the procedure. To date, 13 clinical studies have been published reporting results on over 1300 hands in over 1000 patients at up to 2 years post-treatment comparing recovery time, effectiveness, and safety in subjects with CTS treated with CTR-US or mOCTR.^[20–32]^ Among these over 1300 hands, there were no major neurovascular complications, and the clinical success rate was over 95%. Furthermore, 2 randomized controlled trials and 1 prospective non-randomized trial demonstrated superior early outcomes for CTR-US compared to mOCTR.^[22,25,29]^ However, these trials have been limited by small sample size, short follow-up duration, or both. No randomized controlled trial comparing CTR-US to mOCTR has been performed with a sample size over 100 patients and with at least 1 year of follow-up. Thus, the objective of this randomized controlled trial is to compare the safety and effectiveness of CTR-US versus mOCTR in a large cohort of subjects (n = 120) with symptomatic CTS followed for 1 year post-treatment.

## 2. Design and Methods

This paper describes the rationale and design of TUTOR (Trial of Ultrasound guided CTR versus Traditional Open Release). The protocol was developed in accordance with the SPIRIT 2013 guidance for protocols of clinical trials.^[33]^

### 2.1. Study design

This is a prospective, multicenter, randomized controlled trial that will be performed at up to 12 sites in the United States. Subject recruitment in the study began July 26, 2022. A total of 120 subjects will be enrolled and randomized (2:1) to receive CTR-US or mOCTR. The total study duration is expected to be approximately 1.5 years, with 6 months of anticipated subject recruitment and 1 year of follow-up. The trial was prospectively registered at ClinicalTrials.gov (NCT05405218) before first subject enrollment. The trial was funded by Sonex Health, Inc. (Eagan, MN) who was involved in trial design, but will not be involved in data analysis or publication of trial results. Ongoing trial oversight will be provided by a data safety monitoring board (DSMB) and data will be routinely monitored for accuracy. A list of investigational sites and trial oversight committees is provided in Table 1.

**Table 1:** List of investigators and oversight committees in the TUTOR trial.

Name	Institution *	Location
**Site investigators**		
Kyle R. Eberlin, MD **	Massachusetts General Hospital	Boston, MA
Christopher J. Dy, MD, MPH, FACS	Washington University	St. Louis, MO
James F. Watt, DO	Orthopedic Associates	Fort Walton, FL
James L. Gluck, MD	Kansas Orthopaedic Center	Wichita, KS
Alexander Palmer, DO	Sano Orthopedics	Lee’s Summit, MO
F. Thomas D. Kaplan, MD, FAAOS	Indiana Hand to Shoulder	Indianapolis, IN
Thomas J. McDonald, MD	Sierra Orthopedic Institute	Sonora, CA
Mark D. Fischer, MD	Twin Cities Orthopedics	Maple Grove, MN
Marc E. Walker, MD, MBA	University of Mississippi Medical Center	Jackson, MS
**Data safety monitoring board**		
Kevin C. Chung, MD, MS	University of Michigan Health	Ann Arbor, MI
Julie E. Adams, MD	University of Tennessee College of Medicine	Chattanooga, TN
Warren C. Hammert, DDS, MD	Duke University	Durham, NC
**Independent medical reviewer**		
Kevin C. Chung, MD, MS	University of Michigan Health	Ann Arbor, MI

TUTOR = Trial of Ultrasound guided carpal tunnel release versus Traditional Open Release

* Up to 12 investigational sites may participate in this study. The list of sites in the table represents those that were active as of September 12, 2022.

** Study principal investigator.

### 2.2. Participants and eligibility criteria

Study participants will undergo a preoperative clinical examination and diagnostic ultrasound of the median nerve. Key eligibility criteria of the trial are a clinical diagnosis of unilateral or bilateral idiopathic CTS, a score of 12 or greater on the CTS-6 questionnaire in the target hand,^34^ median nerve cross-sectional area ≥10 mm^2^ in the proximal carpal tunnel region of the target hand,^34^ absence of carpal tunnel symptoms in the contralateral hand that interfere with work or daily activities, and prior failure of nonsurgical CTS treatment. Key exclusion criteria are previous surgery on the target hand or wrist, recent (<6 weeks) corticosteroid injection in the target hand or wrist, need for additional operative procedure, and planned surgical or interventional procedure on the contralateral wrist or hand. Subjects who meet all preoperative eligibility criteria will be randomized to receive CTR-US or mOCTR. A complete list of study eligibility criteria is provided in Table 2.

**Table 2:** Subject eligibility criteria.

Inclusion Criteria
1. ≥18 yrs of age
2. Clinical diagnosis of unilateral or bilateral idiopathic CTS
3. CTS-6 score >12 in target hand
4. Absence of carpal tunnel symptoms in the contralateral hand that interfere with normal daily activities or work at the time of consent and are not anticipated to interfere with return to activities or return to work within at least 3 mo post-operatively
5. Median nerve cross-sectional area ≥10 mm^2^ in the proximal carpal tunnel region of the target hand measured by diagnostic ultrasound
6. Prior failure of one or more nonsurgical treatment options for the target hand (e.g., physical activity modification, bracing, splinting, corticosteroid injection)
7. Subject agrees to complete follow-up questionnaires over a 12-mo period
8. Subject has a valid mobile phone number and email address to receive and answer follow-up questionnaires
Exclusion Criteria
1. Prior surgery on the target wrist or hand with the exception of trigger finger that has clinically recovered
2. History of prior surgical CTR in the target hand
3. History of prior surgical CTR in the contralateral hand within 3 mo of enrollment or with persistent symptoms that interfere with normal daily activities or work at the time of consent
4. Corticosteroid injection in the target wrist or hand within 6 wks of randomization
5. Presence of additional process in the target wrist or hand requiring additional intervention beyond carpal tunnel release (e.g., neurolysis, mass removal, tenosynovectomy)
6. Clinically significant degenerative arthritis of the upper limb (shoulder to hand) on the target side
7. Clinically significant inflammatory disease (including tenosynovitis) of the upper limb (shoulder to hand) on the target side
8. Clinically significant trauma or deformity of the upper limb (shoulder to hand) on the target side
9. Clinically significant vascular disease (including Raynaud’s phenomenon) of the upper limb (shoulder to hand) on the target side
10. Clinically significant neurological disorder (including complex regional pain syndrome) of the upper limb (shoulder to hand) on the target side
11. Planned surgical or interventional procedure on the contralateral wrist or hand
12. Systemic inflammatory disease (e.g., rheumatoid arthritis, lupus)
13. Amyloidosis
14. Chronic renal insufficiency requiring dialysis
15. Diabetes not controlled by a stable dose of medication over the past 3 mo
16. Uncontrolled thyroid disease
17. Pregnant or planning pregnancy in the next 12 mo
18. Workers compensation subjects
19. Inability to provide a legally acceptable Informed Consent Form and/or comply with all follow-up requirements
20. Subject has other medical, social or psychological conditions that, in the opinion of the investigator, preclude them from receiving the pretreatment, required treatment, and post-treatment procedures and evaluations

### 2.3. Randomization

The randomization sequence for this trial was developed by an independent biostatistician and computer-generated by an electronic data capture system (Viedoc, Philadelphia, PA). A 2:1 (CTR-US: mOCTR) randomization ratio will be utilized with the randomization sequence stratified by site using variable block sizes to minimize treatment allocation predictability. Treatment assignment will be concealed until it is presented to authorized site personnel at the time of randomization.

### 2.4. Blinding

Because of obvious differences in surgical technique, it is not possible to blind the treating physicians. As the result of notable visual differences in the postoperative scar between surgical techniques (~3–5 mm for CTR-US and ~1–3 cm for mOCTR), it is not possible to blind the subjects. Due to the fact that the majority of data collected in this trial will be subject-reported, it is not feasible to blind outcome assessors (i.e., subjects).

### 2.5. Surgical procedure

Subjects randomized to CTR-US will be treated with the commercially available UltraGuideCTR (Sonex Health, Inc., Eagan, MN). The device is a single-use, hand-held device that is inserted into the carpal tunnel through a small (typically < 5 mm) incision at the proximal wrist using real-time ultrasound guidance. The working tip of the UltraGuideCTR consists of 2 inflatable balloons that border a centrally located, retractable retrograde cutting blade. Ultrasound is used to position the tip inferior and distal to the TCL and the balloons are inflated with sterile saline, increasing the tip diameter to 8 mm. The inflated balloons displace the median nerve and ulnar artery away from the device, with safe position verified with ultrasound. The blade is then activated, and the TCL is transected in a retrograde manner, with ultrasound visualizing the transection and verifying safe position of the neurovascular structures. Following TCL transection, the blade is recessed, the balloons are deflated, and the device is removed. The TCL is then probed to ensure a complete release. In subjects randomized to mOCTR, the TCL will be divided through a 1 to 3 cm incision in standard fashion without ultrasound guidance. Postoperative patient care instructions will be standardized for each treatment group and among all participating sites in order to minimize bias. Investigators will instruct subjects to participate in activities and return to work, as tolerated, based on pain, function, and wound healing status.

### 2.6. Outcomes

Subject data will be recorded using electronic case report forms and will be routinely monitored for accuracy. Follow-up assessments will occur daily for the first 14 post-procedure days, and at 4 weeks, 3 months, 6 months, and 1 year post-treatment thereafter. Time to return to normal activities and return to work will be assessed daily. Boston Carpal Tunnel Questionnaire Symptom Severity Scale (BCTQ-SSS) and Functional Status Scale (BCTQ-FSS), Numeric Pain Scale, EuroQoL-5 Dimension 5-Level (EQ-5D-5L), and adverse events (AEs) will be assessed at each follow-up interval. A schedule of subject assessments during the study is provided in Table 3.

**Table 3:** Study assessments at each follow-up interval.

Assessment	Baseline	Procedure	Post-Op	Daily 1-14	1 mo	3 mo	6 mo	12 mo
**Site Assessments**								
Demographics	**■**							
Ultrasound median nerve cross-sectional measurement	**■**							
CTS-6 (both hands)	**■**							
Randomization	**■**							
Procedure		**■**						
Image of incision		**■**						
Adverse events		**■**	**■**	**■**	**■**	**■**	**■**	**■**
**Subject-reported Outcomes**								
Demographics	**■**							
Medical history	**■**							
BCTQ-SSS	**■**			**■** *	**■**	**■**	**■**	**■**
BCTQ-FSS	**■**			**■** *	**■**	**■**	**■**	**■**
EQ-5D-5L	**■**			**■** *	**■**	**■**	**■**	**■**
Numeric Pain Scale	**■**	**■**	**■**	**■**	**■**	**■**	**■**	**■**
Procedure		**■**						
Images of wound healing				**■** **				
Return to activities				**■**	**■**	**■**	**■**	**■**
Return to work				**■**	**■**	**■**	**■**	**■**
Pain medication	**■**	**■**	**■**	**■**	**■**	**■**	**■**	**■**

* Collected at 14-day evaluation only.

** Collected at 7-day and 14-day evaluation only.

BCTQ-FSS = Boston Carpal Tunnel Questionnaire Functional Status Scale, BCTQ-SSS = Boston Carpal Tunnel Questionnaire Symptom Severity Scale; EQ-5D-5L, EuroQoL-5 Dimension 5-Level.

The primary endpoint of the study is the percentage of patients who return to normal daily activities within 3 days of the procedure, irrespective of work status. Secondary endpoints of the study are median time to return to normal daily activities, percentage of patients who return to work within 3 days of the procedure, median time to return to work, BCTQ-SSS change score at 3 months, BCTQ-FSS change score at 3 months, Numeric Pain Scale change score at 3 months, EQ-5D-5L change score at 3 months, and the incidence of device- or procedure-related AEs at 3 months. The 3-month follow-up interval was selected for analysis of secondary endpoints because the majority of clinical improvement after CTR occurs in the first 3 months, with marginal improvement thereafter.^[35,36]^

Among study subjects who report full-time or part-time employment preoperatively, time to return to work is defined as the number of days between treatment and the time the subject reports returning to work in any capacity. The BCTQ is a CTS-specific questionnaire that is highly reproducible, internally consistent, valid, and responsive to clinical change in CTS and subject status post-CTR.^[37]^ The BCTQ consists of 11 symptom severity questions (BCTQ-SSS) that are scored from 1 to 5, with higher scores indicating more severe symptoms, and is calculated as the mean of each response. The BCTQ additionally consists of 8 functional status questions that are scored from 1 to 5, with higher scores indicating more functional limitation, and is calculated as the mean of each response. Subjects will be asked to rate their wrist pain severity on a Numeric Pain Scale ranging from 0 to 10, where 0 represents “no pain” and 10 represents “the worst pain imaginable.” The EQ-5D-5L is a generic preference-based questionnaire that measures quality of life across 5 domains: mobility, self-care, usual activities, pain/discomfort, and anxiety/depression. Each dimension is scored on a 5-level severity ranking consisting of: no problems, slight problems, moderate problems, severe problems, unable to/extreme problems.^[38]^

Subject safety will be assessed by recording AEs. An AE is defined as any adverse change (i.e., *de novo* or preexisting condition) from the subject’s baseline medical condition occurring after the initial procedural incision has been initiated. Determination of whether a subject experienced an AE can be made in 3 different ways. First, an AE can be documented by the site during the study procedure. Second, a subject may report a postoperative AE directly via phone call to the investigative site. Third, an AE can be identified by the site during the review of the subject-uploaded wound healing images. If the site identifies a potential AE based on image review or is notified of a potential AE by the subject, confirmation of the AE will occur by a phone call with the subject or, if necessary, by asking the subject to return for a follow-up clinical evaluation.

Adverse events will be classified by seriousness and relationship to the device or procedure. A serious adverse event is defined as one that suggests a significant hazard or side effect, regardless of the relationship to the device or procedure. This includes, but may not be limited to, any event that results in death; is life threatening or places the participant at immediate risk of death from the event as it occurred; requires or prolongs hospitalization; causes persistent or significant disability or incapacity; results in congenital anomalies or birth defects; or is another condition which investigators judge to represent significant hazards. A device-related AE is directly attributable to the device or to improper use of the device. A procedure-related AE is directly attributable to the procedure, irrespective of the device, including complications from anesthesia or other procedures incidental to CTR. The relationship of the AE to the device or procedure will be determined by the site investigator using the following definitions:

- ***Definite*:** The AE follows a reasonable temporal sequence from the time of the index procedure, which includes AEs that occur during the index procedure or during the follow-up period.
- ***Probable***: The AE follows a reasonable temporal sequence from the time of the index procedure, and the possibility can be excluded that factors other than the index procedure, such as underlying disease, concomitant drugs, or concurrent treatment caused the AE.
- ***Possible***: The AE follows a reasonable temporal sequence from the time of the index procedure and the possibility of index procedure involvement cannot be excluded. However, other factors such as underlying disease, concomitant medications, or concurrent treatment are presumable.
- ***Unlikely***: The AE has an improbable temporal sequence from the time of the index procedure, or such AE can be reasonably explained by other factors, including underlying disease, concomitant medication, or concurrent treatment.
- ***Not related***: The AE has no temporal sequence from the time of the index procedure, or it can be explained by other factors, including underlying disease, concomitant medication, or concurrent treatment.

Evaluation and adjudication of all AEs will be performed on an ongoing basis by an independent medical reviewer. The independent medical reviewer will review AEs for AE classification, seriousness, and relationship to the device or procedure. Discrepancies between the investigational site and the independent medical reviewer will be handled by discussion, with the determination of the independent medical reviewer serving as the final classification.

A DSMB will oversee enrollment and safety of the study subjects, and will advise the study sponsor to continue the trial with no modification, or to modify the trial as appropriate if enrollment or safety concerns are identified. Members of the DSMB will be independent of the sponsor and investigational sites.

### 2.7. Statistical analysis

A sample size of 120 subjects was calculated by assuming the percentage of subjects who would return to normal daily activities within 3 days of the procedure would be 60% in the CTR-US group and 25% in the mOCTR group, 2:1 (CTR-US: mOCTR) randomization ratio, and no more than 15% subject attrition using a Fisher’s exact test with a 2-tailed alpha level of 0.05 and 90% statistical power.

All statistical analyses will be performed by an independent biostatistician. The analysis population of this trial will consist of all randomized subjects who receive their assigned treatment. Baseline data will be analyzed using descriptive statistics. Continuous data will be summarized using mean and standard deviation for normally distributed data, median and interquartile range for non-normally distributed data, and counts and percentages for categorical data. For categorical variables, percentages will be calculated based on non-missing data.

The primary endpoint will be reported as the percentage of patients in each treatment group who return to normal daily activities within 3 days of the procedure. Time to return to normal daily activities and time to return to work among employed individuals will be reported as the median and interquartile range in each treatment group. Due to the likelihood that the data distribution will likely be positively skewed, the nonparametric Mann–Whitney *U* test will be the statistical test used to assess these endpoints. Differences between the CTR-US and mOCTR groups with respect to longitudinally measured continuous outcomes (i.e., BCTQ-SSS, BCTQ-FSS, Numeric Pain Scale, EQ-5D-5L) will be analyzed using mixed model analysis and adjusting for the baseline score. Adverse events will be reported using counts, percentages and exact 95% confidence intervals using Clopper-Pearson’s method. The incidence of AEs in each group will be calculated on a per-subject basis and compared using Fisher’s exact test. Statistical analyses will be performed using a 2-sided hypothesis test at a 5% level of significance. No adjustments for multiplicity are planned. Missing data imputation will not be performed.

### 2.8. Ethics and dissemination

The protocol for this clinical trial was approved by a central institutional review board (WCG IRB, Puyallup, WA) and all enrolled subjects will provide informed consent before study participation. The authors intend to report the results of this trial at medical conferences and peer-reviewed journals.

## 3. Discussion

CTR is a common surgical procedure that can be performed using standard open, mini-open, limited incision, endoscopic, or ultrasound-guided techniques. The results derived from TUTOR will fill an important research gap because there is currently limited evidence directly comparing the safety and effectiveness of CTR-US and mOCTR. Major strengths of the current trial include generation of Level 1 comparative evidence, a large sample size derived from multiple investigative sites, long-term follow-up, and rigorous study oversight by an independent medical reviewer and a DSMB. The initial 3-month results of this trial, including assessment of the return to normal daily activities primary endpoint, are planned to be reported in mid-2023. The outcomes of TUTOR will have important clinical and economic implications for all stakeholders involved in treating patients with CTS.

## Acknowledgments

The authors had no writing assistance in the preparation of this manuscript.

## Author contributions

**Investigation:** Kyle R. Eberlin, Christopher J. Dy, Mark D. Fischer, James L. Gluck, F. Thomas D. Kaplan, Thomas J. McDonald, Larry E. Miller, Alexander Palmer, Marc E. Walker, James F. Watt.

**Methodology:** Kyle R. Eberlin, Christopher J. Dy, Mark D. Fischer, James L. Gluck, F. Thomas D. Kaplan, Thomas J. McDonald, Larry E. Miller, Alexander Palmer, Marc E. Walker, James F. Watt.

**Writing – review & editing:** Kyle R. Eberlin, Christopher J. Dy, Mark D. Fischer, James L. Gluck, F. Thomas D. Kaplan, Thomas J. McDonald, Larry E. Miller, Alexander Palmer, Marc E. Walker, James F. Watt.
